# A9 SIALIC ACID METABOLISM PLAYS A KEY ROLE IN INTESTINAL FITNESS AND VIRULENCE OF AN ATTACHING/ EFFACING BACTERIAL PATHOGEN

**DOI:** 10.1093/jcag/gwab049.008

**Published:** 2022-02-21

**Authors:** Q Liang, J M Allaire, H Yu, S M Crowley, X Han, B Vallance

**Affiliations:** 1 Experimental Medicine, University of British Columbia, Vancouver, BC, Canada; 2 BC Children’s Hospital, Vancouver, BC, Canada

## Abstract

**Background:**

The gastrointestinal (GI) mucus barrier acts as an important interface between the host and luminal gut microbes, beyond its role in limiting direct contact between noxious luminal agents and the underlying intestinal epithelium. Mucus is comprised of highly glycosylated mucin proteins, modified by O-glycan side chains, formed by five sugar monomers, including sialic acid. Sialic acid frequently occupies the terminal position of O-glycans and can be cleaved by microbial sialidases. We hypothesize that upon entering their hosts, enteric pathogens, such as the attaching and effacing (A/E) family (EHEC, EPEC and *Citrobacter rodentium*) metabolically adapt to their intestinal environment, and express key virulence factors by sensing and metabolizing mucin sugars, such as sialic acid.

**Aims:**

Investigate the role of sialic acid in regulating the fitness and virulence strategies of the A/E pathogen *C. rodentium* within the GI environment.

**Methods:**

C57Bl/6 mice were orally infected with either wildtype (WT) or mutant strains of *C. rodentium* to study bacterial pathogenicity *in vivo*. Sialic acid was localized in mouse colonic tissue sections through lectin staining, and quantified in mouse feces and mucus scrapings using a commercial kit. Protein secretion by *C. rodentium*, in the presence or absence of sialic acid was analyzed by SDS-PAGE and mass spectrometry. Intestinal epithelial cell lines were infected with enteropathogenic *E. coli* (EPEC) or *C. rodentium* to examine bacterial adherence.

**Results:**

Sialic acid was expressed widely in the GI tracts of mice, primarily in the colonic mucus layer and by intestinal goblet cells. Both EPEC and *C. rodentium* were found to take up and metabolize sialic acid through the transporter NanT. A *C. rodentium* strain deficient in sialic acid uptake ( *ΔnanT*) was dramatically impaired in colonizing the intestines of mice and was rapidly cleared. Sialic acid also impacted *C. rodentium*’s virulence by inducing the secretion of two key virulence factors, which significantly enhanced the pathogen’s adhesion to intestinal epithelial cells. Moreover, sialic acid increased *C. rodentium*’s ability to degrade mucus, due to the increased production of these two secreted virulence factors.

**Conclusions:**

We demonstrate that sialic acid, a mucin-derived sugar, is an essential nutrient for A/E pathogens to thrive and expand within their host’s intestines. Moreover, sialic acid enhances pathogen virulence by inducing secretion of two important virulence factors, which increase adhesion to the epithelium and promote the degradation of mucus.

**Funding Agencies:**

CCC, CIHRCH.I.L.D. Fdn

